# Supplementary material for: A Bayesian non-parametric mixed-effects model of microbial growth curves
Source: PLoS Comput Biol. 2020 Oct 26;16(10):e1008366. doi: 10.1371/journal.pcbi.1008366 (PMC7644099; doi:10.1371/journal.pcbi.1008366)
Supplement: Attachment — Submitted filename: 2020-08-10-phenom-review-response.pdf [file pcbi.1008366.s010.pdf]

Response to reviews, PCOMPBIO-D-20-00079

August 10, 2020

Summary of revisions

Thank you for a thorough and helpful review of our manuscript. In this revision, we have addressed each point raised by the reviewers. Manuscript changes include: (a) removing the preproccessing step discussed by reviewer 2; (b) improving the definitions and clarity of terms throughout; and (c) clarifying the interpretation and purpose of the modeling approach we have developed. These changes are highlighted in red text in the revised manuscript. These revisions have improved upon our original conclusions, which have changed after re-modeling the non-preprocessed data. Namely, this highlights an additional source of technical variation that can be captured by our model, strengthening the motivation for our approach. Below please find our point-by-point responses, with reviewer comments in italics and our responses in plain text.

Reviewer 1

Summary

The authors present a new Bayesian modeling approach for cell growth that is adaptable, allows for incorporation of several effectors, and includes batch-to-batch variation. While there is novelty to the overall approach, the manuscript is difficult to read and the implementation seems convoluted. If these issues can be addressed, the approach described could be adopted widely.

We thank the reviewer for the constructive comments, and have edited the text to improve the legibility of the manuscript as described throughout our responses to all reviewers.

Major Points

Reviewer 1: Comment 1: *Overall, I found the modeling approach intriguing but the manuscript somewhat difficult to read. This is unfortunate given that growth models of microbes should be a topic accessible by a wide audience.*

We have made efforts to clarify the manuscript text throughout, with a specific focus on clarifying in response to particular suggestions from all reviewers, for example, defining terms, variables, and so forth. Please see the red text throughout in the revised manuscript. From our perspective, this significantly improves the readability of the manuscript, and hope that the reviewer will agree.

Reviewer 1: Comment 2: *The modeling approach described here has the potential to be adopted somewhat widely, as many engineers and biologists are looking for better models to describe microbial growth. The ability to incorporate batch-to-batch effects is certainly a benefit, and the ability to add in additional effects, inhibitors, etc. into the framework may make this approach universally adaptable.*

We appreciate the positive feedback.

Reviewer 1: Comment 3: *With that, there are problems that should be addressed. Currently, it is not clear how one would apply this modeling framework to build a model for monitoring bioprocesses.*

The question regarding bioprocesses is an excellent one, as this is a potential area where our models could find broad applicability. However, in the current manuscript we focus on data from microplate readers and not chemostat fermentors, so future application of our model by those in the bioprocess field who generate data from such growth instruments would be welcome and exciting. We have added a note regarding the potential for bioprocess applications in the discussion section (lines 316-317). Given the length of the current manuscript, we feel a proper treatment of this application must necessarily be left to future work.

Reviewer 1: Comment 4: *What phenomics experiments should be run a priori?*

Our model is specifically designed to analyze microbial growth curves. To clarify this point, we have edited the title, which now reads, "A Bayesian Non-parametric Mixed-Effects Model of Microbial Growth Curves". This clarification in the title was also requested by one of the other reviewers.

Reviewer 1: Comment 5: *How are model parameters found?*

Model parameters are obtained using Bayesian statistical inference as described in Section 4.3.4. A more detailed description has been added to Section 4.3.4 to make this clear, and this has also been stated more explicitly in the introduction of the model (Section 2.2, lines 184-185). Calculation of the posterior distribution is performed by use of (Hamiltonian) Markov chain Monte Carlo (MCMC) sampling to perform Monte Carlo integration, using the Stan library, as described in Section 4.3.4.

Reviewer 1: Comment 6: *How many batches and replicates are needed? Is batch-to-batch variability reduced by including more variables in the model?*

This is a very important question that many microbiologists would want to know in designing their experiments. In experiments conducted here, we have included three biological replicates (i.e. cultures inoculated from three different colonies on the same petri plate), three technical replicates (separate inoculations from the same colony), and two batches. However, as discussed in the manuscript (lines 280-284 of the revised manuscript), the optimal experimental design depends on the extent of technical and biological variability present in context of a particular biological study and associated treatment conditions. The ability to use *phenom* for formal statistical experimental design calculations is one of the areas of future work about which we are most excited; however, a

detailed exploration of experimental design with the *phenom* model is beyond the scope of the current paper. As we suggest in Section 2.4, this would involve running *phenom* iteratively with high throughput growth curve experiments, i.e. running *phenom* on a pilot dataset to provide estimates of variance component parameters and then determination of sample (batch and replicate) sizes based on the desired error tolerance. We have clarified this point in Section 2.4 (lines 285-289).

Reviewer 1: Comment 7: *The code is available through GitHub, but the implementation in Python is not for the inexperienced user. From this, my main suggestion is to improve accessibility and provide user-friendly instructions and guidelines. This could lead to a large number of citations.*

We have updated both the readme of the library repository (github.com/ptonner/phenom) and improved the example jupyter notebook to better represent the workflow for applying *phenom*. Additionally, we will be collaborating this fall with graduate students in the Duke University Masters in Interdisciplinary Data Science program to further improve the usability of this software for a broader audience.

Reviewer 1: Comment 8: *The introduction does not mention the contribution of "genome-scale" kinetic and dynamic flux-based modeling strategies in determining growth rates, effects of inhibitors, multiple substrates, genetic perturbations, etc. There is a wealth of knowledge in the literature that should be referenced. How does the Bayesian modeling approach proposed in this paper compare to the other dynamic genome-scale approaches that try to incorporate mechanistic effects?*

The reviewer references to an important, large body of literature on constraint-based approaches such as flux balance analysis (FBA). However, *phenom* is an *empirical* model for microbial growth data (e.g. optical density) appropriate for statistical analysis of data sets consisting of experimental growth curves. It does not attempt to provide a mechanistic model for the molecular or cellular factors contributing to growth changes, but rather to model the *data* generated by a given set of experiments. As with most statistical data analysis methods, it is therefore able to account only for those effects incorporated into the experimental design (e.g mutations, stress conditions, etc.). The use of *phenom* to interrogate mechanistic effects therefore requires appropriately designed experiments to compare mechanistic hypotheses, in experiments where the observable outcome is growth curve data. In such situations, *phenom* would be the preferred tool for determining the significance of the difference between curves. *Prediction* of growth curves is not the goal of *phenom*, precisely because it is a flexible non-parametric statistical model for analyzing and interpreting data, rather than an theoretical mechanistic model for predicting from first principles.

In contrast, FBA and other genome-scale constraint-based approaches predict growth rate based on a detailed mechanistic model incorporating (ideally) the total set of reactions in the metabolic network of an organism. We consider these types of methods to be quite different from *phenom* in their purpose, their approach, and their applicability. For this reason, from our perspective the flux-based modeling literature is not relevant here. There may be potential to incorporate information from these models in future versions of *phenom*, but this remains out of the scope of the current paper.

Reviewer 1: Comment 9: *Eq. 6 contains several conditional statements, and these can be expanded to incorporate other conditions, I'm assuming. How is this different from other parametric Monod-type models that include terms for inhibition, multiple substrates, etc.? Don't they all contain constants that must be found through experimental data? Can't a Monod-type model contain a batch-to-batch variability parameter? An effective approach would be to compare several different model types given the same experimental data generated in this study. There is some reference to previously published studies in this regard, but the findings remain unclear. It seems some direct comparisons can be made in this manuscript.*

Monod-type parametric models fit a sigmoid function to estimate maximum instantaneous lag phase, growth rate (μ_{max}), and carrying capacity (A) (1; 2). We agree with the reviewer that these can be expanded to include additional variables, but the underlying assumption of a sigmoid function remains. This assumption is a fundamental problem when dealing with non-standard growth conditions (stress response, diauxic growth, shock response, etc.), which deviate substantially from the sigmoid form. We have shown this previously in the referenced papers (2; 3) and we have added further emphasis to this point in the Introduction section of the revised manuscript (lines 49-50). In addition, the parameters in the Gaussian Process *phenom* model represent curves that flexibly learn the growth curve from the data, rather than constants. We appreciate the suggestion to compare *phenom* to parametric models, which was included in the previous version of the manuscript (Figures S3 and S4, explained in detail on lines 128-147 of the main text of the revised manuscript).

Reviewer 1: Comment 10: *It's not clear why a single batch can contain multiple growth curves. Are these all of the same medium lot? All of the curves seem to be related even though they were grown separately. A batch is usually from a single fermenter. I'm not sure how to address this better, but it is another somewhat confusing aspect in the manuscript.*

Here we use the term "batch" to refer to a single run of the microplate reader, as we described on line 105. However, we appreciate the reviewer's concern that we are using the term "batch" in a different sense than is typically used in the field of bioprocess engineering. We have therefore included additional emphasis around this definition on lines 105-108 of the revised manuscript.

Reviewer 2

Summary

Reviewer 2: *This manuscript by Tonner et al. describes a much-needed contribution to the rigorous modeling of bacterial growth. They extend their previous work on Bayesian modeling of microbial growth curves by creating a fully non-parametric framework that accounts for experimental and technical variability. They demonstrate how incorporation of batch modeling terms can change interpretation of results from growth experiments performed in varying media conditions for* P. aeruginosa *and* H. salinarum*. Overall, the manuscript is very well written and logically builds on previous work. This approach will advance studies of microbial growth curves, most of which currently apply heuristic methods for summarizing growth curves into a single metric (growth rate, AUC, etc.). I have essentially no concerns related to the utility or implementation of the phenom*

method, but highlight one issue with the data source for P. aeruginosa *that warrants reprocessing and reanalysis. I will use the methods the authors have developed in my own projects and I am very enthusiastic about the line of research being developed by the manuscript authors.*

We thank the reviewer for the positive comments. We also appreciate the time and effort this reviewer has taken to re-run our code, catching the redundant preprocessing step in the *P. aeruginosa* data; we address this below.

Major Comments

Reviewer 2: Comment 1: *Unrealistic values of log(OD) are plotted in most figures describing growth of* P. aeruginosa *in the manuscript. One concrete demonstration of the problem is in the* P. aeruginosa *plots in Figure 1B; log(OD) = 5 is reported in many panels. Even assuming a base of 2, that is a raw OD of 30, an order of magnitude higher than the maximum values that might be expected in very favorable growth conditions. What is the base of the logarithm? In digging through reference 23, it was reported that raw OD values were log2 transformed; however, the raw values in the github repository for that paper are between around 0-1.5 yet the processed data are in the range of around 0-6. In the paper itself, all plotted OD values seem to be from the raw data rather than processed data (or a different version of the processed data). In the publication's github repo, it looks like there may be a problem with the scaling process (https://github.com/amyschmid/pseudomonas-organic-acids/blob/master/preprocess.ipynb).*

*Specifically, the problem seems to be in this loop: for k,index in group.groups.items(): temp = data.loc[:,index] od = temp.values[:5,:].ravel() coeff = np.polyfit(time.tolist()*temp.shape[1],od,2) temp = temp - np.polyval(coeff,data.index.values[0])*

A second order polynomial is fit to the first 5 time points, and then the polynomial is evaluated at the first time point. This results in subtraction of a large negative number (-6.17 for the example growth curve I used), inflating the OD for the entire growth curve. The motivation for the polynomial fitting procedure on only the first five time points, then the scaling by the polynomial fit value for only the first time point, is unclear to me. Why not remove the first five time points, and scale the entire curve by subtracting by the real value of the first time point? The choice to scale based on a within-sample polynomial fit may also introduce artificial technical variation between samples.

Please resolve this issue with the data, rerun all analyses for P. aeruginosa, and include the base of the logarithm in the axis titles or the figure legend. I do not think that this issue points to any underlying problems with the phenom method. The H. salinarum data do not raise any red flags for me, but I would encourage you to inspect the data with the same rigor that you should for P. aeruginosa, given the potential errors.

This is an important point and we thank the reviewer for highlighting this issue. The reviewer correctly re-constructed the pre-processing steps that we took on the growth data for both *Hbt. salinarum* and *P. aeruginosa*, with one additional detail that normalization was grouped within a specific experimental design (e.g. standard growth or oxidative stress) when computing the normalization factor. This pre-processing step, which was applied in previous analyses (2; 3), was employed to control for observed deviations between treatment and control effects that occur in the initial (time zero) growth measurements that are typically due to technical variation — for example, in growth state between starter cultures, or instrument variability in the first few OD readings.

These lead to an artificial offset between conditions. This can lead to estimated differences in growth measurements at time zero, where generally no effect is expected. However, when making the data transformation during pre-processing, the correct scale is no longer the logarithm of optical density, but the logarithm of *fold-change*. As the reviewer notes, the values reported as log OD are infeasible, which is due to this transformation shifting all values by a large value (e.g. around 6 for the *P. aeruginosa* data). We regret the error in reporting these values as log OD, and thank the reviewer for pointing out this discrepancy. Next, we discuss how we addressed this issue.

As mentioned, this pre-processing step is a remnant of earlier work, whereas the current methodology is fully capable of accomplishing this normalization automatically within the framework of the *phenom* model. As such we have removed this step and re-run all analyses both *Hbt. salinarum* and *P. aeruginosa* on the original raw (non-pre-processed) optical density measurements. The result was that all values *are* now properly reported as $\log$(OD). Indeed, one of the strengths of the current approach is the ability to avoid heuristic preprocessing, which can potentially introduce artifacts into the data. Instead, *phenom* handles the data directly in a way that would match the expectation of microbiologist practitioners in how growth data should be reported and modeled. We found that the majority of conclusions remain unchanged compared to the previous analyses. However, certain novel results from this re-run strengthen the argument for using *phenom* for modeling microbial growth phenotypes. Examples are detailed below.

All figures relating to reported growth measurements (Figs 1, 2, 4, 5) have been updated to present the results on the non-pre-processed optical density measurements. These figures also include other style updates in response to other comments by the reviewers, as listed throughout this document. Fig 1 now represents the OD levels expected for both organisms, for example a maximum of around $\log \text{OD} = 1$ for *P. aeruginosa*. Fig 2 C and D were similarly updated. We note that Fig 2 A and B remain unchanged, as the parametric growth models are typically modeled on the same fold-change scale we used previously (1), but we have updated the axis label for Fig 2A to reflect this.

Fig 4 shows the updated model estimates for both *Hbt. salinarum* and *P. aeruginosa*. Overall, the broad conclusions from this figure remain unchanged. However, it is now clear that there is a prominent increase in variability of treatment effects for *Hbt. salinarum* for both low and high oxidative stress, previously masked by the centered measurements. The *P. aeruginosa* estimates are less influenced due to the fact that we report the derivative of treatment effects, which is less impacted by shifting the data by a fixed value, as happens during pre-processing. Due to the increased variability of *Hbt. salinarum* growth data, we have updated the main text to highlight this effect (lines 216 — 219).

Fig 5 shows the updated treatment effect estimates of all models with the updated measurements. We note that the majority of conclusions from this figure also remain unchanged. However, the updated analysis now better represents the importance of modeling all random effects when analyzing growth data. Specifically, for low oxidative stress, we found that the posterior interval for the treatment effect under M_{full} now is insignificant across the entire growth curve. This represents a stronger claim about the differential impact of modeling batch effects than the previous approach (in the previous Fig 5B). The original Fig 5B was removed, as the posterior interval of M_{null} did not deviate as heavily from the posterior mean of M_{full} with the current measurement data. However, we feel the new results, in which M_{full} does not find any significant treatment effect for low oxidative

stress, better highlight the importance of controlling for batch effects when modeling experimental growth data. We updated the main text to reflect these changes at lines 243—248. We also found that the M_{full} model was capable of correcting for the technical variation seen in treatment effects at time zero. We have added a supplemental figure that discusses this point (main manuscript lines 248-249 and Fig. S7).

Reviewer 2: Comment 2: *Starting around the text describing Figure 4, "significant" starts to be used in a way that isn't defined in the text (line 207, "significantly different posterior estimates", line 220, "... is estimated to be significant during the time points..."; again on lines 221, 222, 224). Please define the procedure used to establish significance in these cases, or use a different term if a formal procedure was not used (which seems completely fair to me given the use of fully probabilistic models).*

We thank the reviewer for pointing out this lack of clarity. Throughout the manuscript, statistical significance is determined formally by calculating 95% posterior credible intervals and determining whether the interval covers zero when assessing a treatment effect (e.g. $\delta(t)$), or determining whether intervals overlap when comparing two estimated quantities. We have added a clarification of this definition to the manuscript (lines 211-214 and 223).

Reviewer 2: Comment 3: *This is not a concern but a strong recommendation: the points about shrinkage of the credible interval with Mfull in Figure 5C are perhaps the most important in the manuscript, but are hard for the reader to grasp because of the overlapping intervals. The point is clear in the text but could be drawn out more clearly in the figure. There are many ways you could emphasize this, but I think the easiest would be having subpanels beneath each panel in Figure 5C which individual plots the estimates of Mnull, Mbatch, and Mfull (e.g., 3 plots with individual models and one plot with estimates from all models shown together for context).*

We thank the reviwer for this suggestion, and have added sub-panels for M_{null} and M_{full} for all plots in Fig 5. We felt that the contrast between these two models gave the best comparison for showing the shrinkage effect of our approach, and as such did not include M_{batch} as an additional subplot.

Minor Concerns

Reviewer 2: Minor concern 1: *Lines 106-112, "technical", "biological", and "batch" should be defined by describing the experiments performed. Based on my own terminology usage, I assume for* H. salinarum *that 9 experiments were performed in which a frozen stock was struck on plates, a colony/lawn was gathered and pre-cultured, and the preculture was standardized via OD and used to inoculate each well ("9 batches"). Within each of these 9 separate experiments, each environmental condition/media condition was replicated in quadruplicate (i.e., "4 biological replicates"), and at each time point OD was measured from in each of the biological replicate wells three times (i.e., "3 technical replicates"). Many other researchers consider the "biological replicates" I describe here to be "technical replicates". We also recommend indicating whether media and/or supplements (e.g., organic acids) were made separately for each "batch", or whether the same solutions were added to media ingredients form the same lot, etc.*

Descriptions of technical and biological replication are given in the introduction on lines 103—105, and there we have added clarifying details to the definition of batch. We have also added clarifying details to the methods section (see first paragraph of section 4.1). In these clarifications, we specify that a "batch" is defined as a single run of the microplate reader, which automatically monitors growth for 200 micro-cultures simultaneously. Each culture grown from a separate colony on a plate streaked from frozen culture represents a "biological replicate". Each biological replicate starter culture was pipetted into three independent wells of the microplate, representing "technical replicates".

Reviewer 2: Minor concern 2: *Line 292; "Cultures were then diluted to OD600 0.05 in a high throughput microplate reader" - was the culture diluted to an OD600 of 0.05 as measured in a 1cm cuvette and then transferred to the microplate, or was culture diluted such that within each well of the microplate the OD600 was 0.05 at the beginning of the growth experiments as measured by the platereader? Please clarify, and if the latter is the case, please indicate the format of the microplates (e.g., 96-well flat-bottom) and the total volume used in each well. Please provide a similar level of detail in the description of culture setup for* P. aeruginosa *as well.*

OD of starter cultures was measured in a 1 cm cuvette, diluted to OD 0.05, then transferred to the microplate reader. We have clarified this point in the main text (lines 324—325). We have also included the matching detail for *P. aeruginosa* (lines 332—333).

Reviewer 2: Minor concern 3: *All figure captions and supplemental figure captions: mu and delta are occasionally expressed as a function of x (instead of t, as in the rest of the manuscript).*

We have corrected this notation throughout.

Reviewer 2: Minor concern 4: *Labelling some panels of Figure 4 with species information would make the figure more interpretable; e.g., having only two batches in panel C makes it tough to figure out that the two colors are still referring to different batches and not some new concept.*

Thank you for the suggestion. We have implemented this change in the revised version of Figure 4. This has enabled consolidation of the figure, which now has only two panels, A and B.

Reviewer 2: Minor concern 5: *In Figure 4C, why is the posterior estimate of the interaction term expressed as the derivative of the growth curve rather than using log(OD) as for all the other estimate plots?*

Derivatives of treatment effects can be estimated directly from the functions already being modeled, as was done for *P. aeruginosa* (shown in Figs. 4B and 5B of the revised manuscript). Reporting derivatives of functional effects can have advantages for interpretation and statistical inference. Namely, the derivative can more accurately reflect differences between curves, and the maximum instantaneous growth rate can be identified visually in the curves. We have reported the derivative of the treatment effects for *P. aeruginosa* growth as an additional example of how to view the estimated effects, which highlights the many uses of *phenom*. We have added clarifying details to the main text on this point (lines 205-207, 256-258), as well as showing the log(OD) curves in the new Fig S8.

Reviewer 2: Minor concern 6: *In Figure 5A, label the left/right panels as low/high OS, respectively, on the figure itself*

We have revised the figure as suggested.

Reviewer 2: Minor concern 7: *Lines 71-73, "The presence. . . " is incomplete, seems like the middle of the sentence should have the following inserted: "..., but [these efforts did not include modeling of] individual batch effects for each term in the model."*

We have corrected the error and edited the sentence as suggested (lines 75-76 of the revised manuscript). Thank you for catching this.

Reviewer 2: Minor Concern 8: *Below are recommendations that we believe would improve the readability of the manuscript but do not think necessary to revise as part of the peer review process.*

The broad readership of PLOS Computational Biology would benefit from explicit definition of several statistical modeling terms used throughout the paper. We recommend defining the following terms at first mention in the introduction. Some of these terms are defined in the text but could use additional clarification. The following are just a few of these terms; it may help to have an application-oriented computational biologist read your manuscript and identify terms that they don't understand which limit their ability to understand the purpose and findings of the paper.

The reviewer makes an excellent point regarding clarity in the definition of terms, and other reviewers had similar concerns. We have incorporated these suggestions by editing the text of the introduction to clarify each term that the reviewer calls out below.

- *fixed effects. This definition seems suitable*
- *random effects. This definition seems suitable but would benefit from using a statistical synonym for "population" since population implies something different in "population growth model". Conversely, "population growth model" could use a biological synonym for "population".* We have changed the use of "population" to "set" when referring to random effects to address this ambiguity (lines 58, 59, 64) and to "distribution" when describing the model (line 175). We have defined "population growth" as the "microbial growth curve" in the introduction (lines 35-36) to clarify usage in the biological sense.
- *parametric model. Clearer indication that the parameters that make standard population growth models "parametric" are those that bake in the assumptions of lag-log-stationary, rather than another assumption about a distribution.* We have added clarification of this point to the introduction (lines 49-50).
- *secondary model. The average reader will not be able to define "secondary model" based on the context provided alone.* We have changed the terminology here to provide better context, changing "secondary model" to "model extension" (lines 51, 54).

Reviewer 3

Summary

Reviewer 3: *This paper presents phenom, a method and software tool for fitting non-parametric mixed-effects models to microbial growth curves. The method is a generalized version of previous work by the same authors with a focus on handling batch and replicate effects without relying on a parametric growth curve. My main concerns are twofold: 1.) are interesting parts of the stress response swept into batch effects, and 2.) do the perturbation functions extracted by phenom correspond to the underlying biology?*

We appreciate these concerns and have addressed them specifically in response to comments below.

Major Comments

Reviewer 3: Comment 1: *My first concern is largely technical. The* phenom *method assumes that batch effects are simply random effects that perturb the true underlying* $\mu(t)$*. As a microbiologist, I'm not sure this is true. Take, for example, the PA growth curves if Fig. 1B with pH 5.5 and 10 mM acid. I have a hard time believing that the* $\mu(t)$ *underlying these curves are the same across the two batches. Bacteria do not have a single response to every combination of stressors. Small (random) perturbations early on could lead to vastly different transcriptional states and therefore two different functions* $\mu(t)$*. My interpretation of these data is the conditions (pH 5.5 and 10 mM acid) are an unstable region of the stress response network. There are actually two* $\mu(t)$*, or at least two functions* $\delta(t)$*, that the organism is pushed into randomly. Said another way, this is not a batch effect but rather a phase transition.*

One way to analyze this is to look at how the function $\delta(t)$ *behaves over the experimental region. Does phenom correctly place a steep transition around pH=5.5 and 10 mM acid? If not, then I would be concerned that the batch effects are hiding large changes in the stress response.*

We thank the reviewer for raising this concern. This is an important question, and we address this by clarifying three specific points below:

- First, it is important to clarify that the Gaussian process model specifies a *distribution* over curves. This means that for a specific combination of stressors, the model would not predict a single curve but an entire distribution (or population) of curves *for the mean behavior*. Thus under our model, a single curve can be obtained by sampling a mean curve from a *distribution* of such curves, and then adding other sources of variability such as experimental noise to obtain the observed curve. Therefore, *phenom* already accounts for the fact that, as the reviewer correctly points out, *"Bacteria do not have a single response to every combination of stressors."* Addition of batch and replicate effects then allows this distribution of curves to shift to accommodate batch- and replicate-specific differences *in the distribution*. Existence of an unstable region of the stress response network, although not modeled here explicitly in a mechanistic way, would therefore manifest in a broadening of the distribution of curves for a given combination of stressors.

- Second, we want to be clear that there is no underlying mechanistic model within *phenom*, it is purely empirical. Please see the response to Reviewer 1, Comment 8 above for a more detailed clarification of this point.
- Finally, it's important to be clear how we interpret "batch effect" in the context of the model. In particular, attributing the observed difference between growth curves to a batch effect does not preclude the existence of a biological effect, such as the example suggested by the reviewer in Fig 1B. Batch effect does, however, indicate that any such biological effect is *completely confounded with the experimental design* in the study analyzed here, and therefore no such biological effect can be confidently inferred based on the current dataset alone. This point is expanded upon more thoroughly below in response to the same reviewer's next point.

Reviewer 3: Comment 2: *My second, and most significant, point is that the presentation of phenom feels disconnected from the underlying biology. As the authors mention, growth curves are rich sources of data regarding phenotypes and stress responses. The main claim of phenom is that it can dissect different sources of variability to uncover the biologically important signal. The paper shows many examples where batch effects supposedly hide perturbations, but there is no ground truth by which we can assess if the uncovered perturbation functions are correct. Many of these data were collected from other papers that study the underlying biology. It is important to show that the differences between the improved models more accurately reflect the stress response.*

For example, the authors note that M_{null} *finds significant perturbations from 10-40 hours, but* M_{batch} *reports the significant interval is 20-40 hours (line 220). Which is correct? Without comparing these intervals to the biological stress response, we cannot say which method is better; we can only say that they are different.*

Ultimately, phenom will only be useful if it can provide insight into the underlying biology. I suggest the authors place a greater emphasis on how well their method translates OD measurements into latent biology. It would be fantastic if the authors can show how phenom could reduce the need for detailed intracellular characterization of the stress response, or at least identify interesting timepoints for further study.

This reviewer concern highlights the need for precise language and motivation when discussing the estimates from models like *phenom*. Below we highlight three main points to clarify the model interpretation.

- First, the reviewer raises the point that the differences seen between treatments of different batches (e.g. PA growth in pH 5.5 and 10 mM citric acid) are likely due to a biological difference between the batches of experiments. We agree that this is a possibility; however, due to the fact that such a biological difference, even if it exists, is completely confounded with batch grouping in the study analyzed, the only robust statistical conclusion to be made from these data is that there appears to be some measurable difference between the growth response to that treatment. Put another way, we do not assert that the effects are nonbiological. Rather, we simply posit that we cannot say with confidence (statistical significance) that there *is* a biological effect, because any such effect is completely confounded with the batching in the design. Additionally, we clarify that with the term "random effect" we do not

mean “spurious”. As the reviewer points out, many biological effects have a stochastic component that are best modeled as random effects. It is the fact that the difference is accounted for by the batch-specific random effect that makes it impossible to conclude a biological signal from these data alone. In this case, modeling batch effects explicitly serves to highlight areas where further study - using a different experimental design - may generate valuable information, e.g. by discovering biological mechanisms driving the differences observed across batches in the current study. To clarify this point, we have expanded our discussion regarding the interpretation of random batch effects with respect to the underlying biology (lines 303—306).

- Second, the reviewer asks whether M_{null} or M_{batch} is correct. In general, some uncertainty will always remain in the magnitude of the treatment effect such that the true effect can never be known exactly. Judging “correctness” of a statistical model is a more complex question, as *phenom* is an empirical and not a mechanistic model: correctness would be best assessed by (complexity normalized) measures of fit to the data. However, as a general principle, it is important that a model properly account for all sources of variability in the measurements. In this case, we have shown that the full model best achieves this, as we believe there is sufficient evidence that: (a) batch effects are present in the data modeled here, as highlighted by the varying estimates of effects for individual experiments (Fig 4); and (b) that these effects are prevalent across microbial growth curve studies for the two different organisms of focus here. Our comparison of M_{null} to M_{batch} and M_{full} are thus primarily intended to highlight the importance of considering these batch effects during statistical inference because the absence of these effects changes the conclusions of modeling (as seen in the differences between M_{null} and the other models). We add clarifying discussion to lines 235—238 to address this point.
- Finally, we agree that the value of these models comes from how they can translate into biological understanding. However, we disagree that this remains to be shown for the modeling approaches presented here and in our previous work (3; 4; 2). We have applied the general fixed effects model to multiple phenomic growth studies to establish how these techniques can guide biological understanding. For example, mapping a transcription factor phenotype network (3), identifying a species-dependent oxidative stress sensitivity across 96 yeast strains (2), and recovering interactions between medically relevant treatment effects not detected by parametric models (4). In the current study, it is our primary goal to establish that these existing techniques, and the downstream interpretation they provide, benefit from considering the batch effects that appear across microbial growth datasets.

Minor Comments

Reviewer 3: Minor Comment 1: *I thought the paper was very well written. It was clear and concise.*

We thank the reviewer for their positive comments.

Reviewer 3: Minor Comment 2: *I suggest changing the title from “microbial phenotypes” to “microbial growth curves”. “Phenotypes” is a broad term that includes almost any measurable feature*

of a microbe, e.g. toxin secretion or sporulation. This paper focuses on only one phenotype, i.e. growth. Growth is a very important phenotype, so I don't believe the more precise title diminishes the work.

We appreciate the suggestion. We agree that the suggested change is clearer, and have revised the title accordingly.

Reviewer 3: Minor Comment 3: *The notation can be confusing, although I believe the confusion stems from notational conventions in two different fields rather than poor choices by the authors. The variable μ is used in microbiology as the instantaneous growth rate of a microbe. The authors use this convention when describing their parametric models (μ_{max}, line 126). Later (line 148) the authors define $\mu(t)$ as the "average growth behavior of an organism". From Figure 3 and eq (1), we see that this definition of $\mu(t)$ implies the units of μ are population size or [OD]. My suggestion is to change $\mu(t)$ to some other name. This may be strange for statisticians, but I believe research focused solely on microbial growth curves should not repurpose the standard names for the parameters in a sigmoid growth curve.*

It is standard in most fields using statistics to denote the mean as μ, and especially so in ANOVA decompositions, hence the choice of $\mu(t)$ for the mean function in functional ANOVA. However, as the reviewer points out, the literature on parametric growth models commonly uses μ_{max} to denote a rate. We have changed the notation for the mean function to $m(t)$ here to remove the possibility of confusion.

References

[1] M. H. Zwietering, I. Jongenburger, F. M. Rombouts, K. van 't Riet, Modeling of the Bacterial Growth Curve, Applied and Environmental Microbiology 56 (6) (1990) 1875–1881.
URL http://www.ncbi.nlm.nih.gov/pmc/articles/PMC184525/

[2] P. D. Tonner, C. L. Darnell, B. E. Engelhardt, A. K. Schmid, Detecting differential growth of microbial populations with Gaussian process regression, Genome Research 27 (2017) 320–333. doi:10.1101/gr.210286.116.

[3] C. L. Darnell, P. D. Tonner, S. Schmidler, A. K. Schmid, Systematic discovery of archaeal transcription factor functions in regulatory networks through quantitative phenotyping analysis, mSystems 2 (2017) e00032–17.

[4] F. M. L. Bushell, P. D. Tonner, S. Jabbari, A. K. Schmid, P. A. Lund, Synergistic impacts of organic acids and ph on growth of *Pseudomonas aeruginosa*: A comparison of parametric and bayesian non-parametric methods to model growth, Frontiers in Microbiology 9 (2019) 3196. doi:10.3389/fmicb.2018.03196.
URL https://www.frontiersin.org/article/10.3389/fmicb.2018.03196
